# Oocyte mitochondria link maternal environment to offspring phenotype

**DOI:** 10.21203/rs.3.rs-4087193/v1

**Published:** 2024-03-29

**Authors:** Jason F. Cooper, Kim Nguyen, Darrick Gates, Emily Wolfrum, Colt Capan, Hyoungjoo Lee, Devia Williams, Chidozie Okoye, Andrew P Wojtovich, Nicholas O. Burton

**Affiliations:** 1 Van Andel Research Institute, Department of Metabolism and Nutritional Programing, Grand Rapids, Michigan, USA, 49503; 2 Van Andel Research Institute, Grand Rapids, Michigan, USA, 49503; 3 University of Rochester Medical Center, Department of Anaesthesiology and Perioperative Medicine, 575 Elmwood Ave., Rochester, NY, 14642, Box 711/604, USA.

**Keywords:** Intergenerational, *C. elegans*, oocyte, mitochondria, AMP kinase, insulin, *isp-1*, *nduf-7*, *nuo-6*, *mev-1*, *gas-1*, *daf-2*, *aak-2*

## Abstract

During maturation oocytes undergo a recently discovered mitochondrial proteome remodeling event in flies^1^, frogs^1^, and humans^2^. This oocyte mitochondrial remodeling, which includes substantial changes in electron transport chain (ETC) subunit abundance^1,2^, is regulated by maternal insulin signaling^1^. Why oocytes undergo mitochondrial remodeling is unknown, with some speculating that it might be an evolutionarily conserved mechanism to protect oocytes from genotoxic damage by reactive oxygen species (ROS)^2^. In *Caenorhabditis elegans*, we previously found that maternal exposure to osmotic stress drives a 50-fold increase in offspring survival in response to future osmotic stress^3^. Like mitochondrial remodeling, we found that this intergenerational adaptation is also regulated by insulin signaling to oocytes^3^. Here, we used proteomics and genetic manipulations to show that insulin signaling to oocytes regulates offspring’s ability to adapt to future stress via a mechanism that depends on ETC composition in maternal oocytes. Specifically, we found that maternally expressed mutant alleles of *nduf-7* (complex I subunit) or *isp-1* (complex III subunit) altered offspring’s response to osmotic stress at hatching independently of offspring genotype. Furthermore, we found that expressing wild-type *isp-1* in germ cells (oocytes) was sufficient to restore offspring’s normal response to osmotic stress. Chemical mutagenesis screens revealed that maternal ETC composition regulates offspring’s response to stress by altering AMP kinase function in offspring which in turn regulates both ATP and glycerol metabolism in response to continued osmotic stress. To our knowledge, these data are the first to show that proper oocyte ETC composition is required to link a mother’s environment to adaptive changes in offspring metabolism. The data also raise the possibility that the reason diverse animals exhibit insulin regulated remodeling of oocyte mitochondria is to tailor offspring metabolism to best match the environment of their mother.

## Results

While diverse animals (including humans) remodel their mitochondrial proteome in oocytes^1,2^, the physiological relevance of this phenomena is unknown. A recent study speculated that ETC remodeling prevents the generation of reactive oxygen species (ROS) that might otherwise cause heritable genetic damage^2^. However, this hypothesis is untested. Studies in flies and frogs have demonstrated that oocyte mitochondrial proteome remodeling is linked to maternal insulin signaling^1^. If mitochondrial proteome remodeling in oocytes exists to prevent ROS-mediated genotoxicity, then it remains unclear why such a process would be linked to an environmentally responsive somatic signaling pathway like insulin signaling and not simply part of normal gametogenesis. Recently, we found that maternal *C. elegans* exposure to osmotic stress leads to a 50-fold increase in offspring survival in response to future osmotic stress via a mechanism that also depends on insulin signaling to oocytes^3^. Since then, reduced insulin signaling to oocytes was similarly found to promote an offspring adaptation to nutrient stress in fruit flies^4^. Based on these findings across different species and for different environmental stressors, we hypothesized that changes in insulin signaling to oocytes might be an evolutionarily conserved mechanism to promote adaptive changes in offspring metabolism that prepare offspring for their mother’s current, external environment. Furthermore, we hypothesized that these effects might be transmitted to offspring by remodeling mitochondrial ETC composition in oocytes.

### Maternal environment alters offspring mitochondrial protein abundance

**Figure 1.**
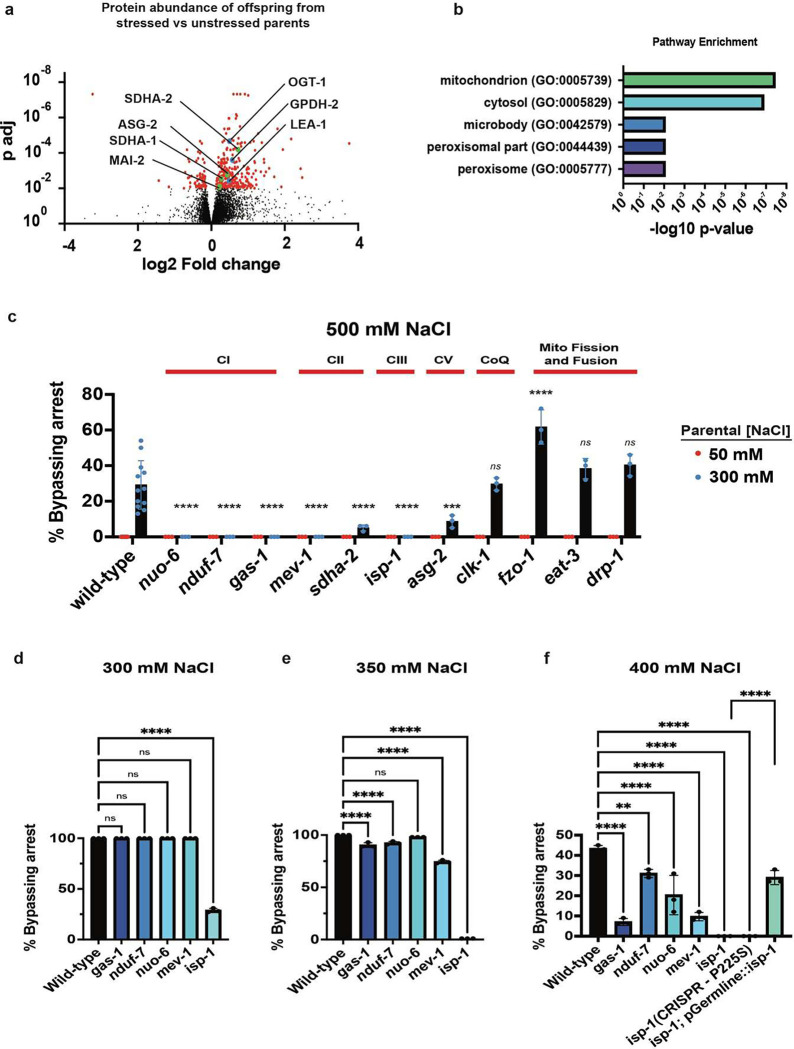
Proper OxPhos function is required for *C. elegans* to intergenerationally adapt to osmotic stress. (a) Volcano plot of 7,161 detected proteins in wild-type *C. elegans* embryos from parents exposed to either 50 mM (normal) or 300 mM NaCl (stressed) for 24 hours. Red dots are the 371 proteins that exhibit significant (*padj* < 0.01) changes in abundance relative to the 50 mM NaCl condition. (b) Cellular components with a significant (*padj* < 0.01) enrichment over expected in the offspring of parents exposed to 300 mM NaCl. Enrichments were determined with WormEnrichr^46,47^. (c) Percent of wild-type, *nuo-6(qm200), nduf-7(et19), gas-1(fc21), mev-1(kn1), sdha-2(tm1420), isp-1(qm150), asg-2(tm1472), clk-1(qm30), fzo-1(tm1133), eat-3(ad426),* and *drp-1(tm1108)* animals developing after 48 hours of exposure to 500 mM NaCl. Red dots represent offspring from parents grown under normal osmotic conditions (50 mM NaCl). Blue dots represent offspring from parents exposed to 300 mM NaCl for 24 hours. *n = 3* replicates of > 40 offspring per replicate. Error bars – s.d. (d) Percent of wild-type, *nuo-6(qm200), nduf-7(et19), gas-1(fc21), mev-1(kn1),* and *isp-1(qm150)* mutants mobile and developing after 48 hours of exposure to 300 mM NaCl. n = 3 replicates of > 100 animals per replicate. Error bars – s.d. (e) Percent of wild-type, *nuo-6(qm200), nduf-7(et19), gas-1(fc21), mev-1(kn1),* and *isp-1(qm150)* mutants developing (compared to those that have entered a state of near suspended animation) after 48 hours of exposure to 350 mM NaCl. n = 3 replicates of > 100 animals per replicate. Error bars – s.d. (f) Percent of wild-type, *nuo-6(qm200), nduf-7(et19), gas-1(fc21), mev-1(kn1),* and *isp-1(qm150)* mutants mobile and developing after 48 hours of exposure to 350 mM NaCl. pGermline refers to animals that express a wild-type copy of *isp-1* under the control of the *pie-1* promoter. CRISPR-P225S represents a CRISPR re-creation of the *qm150* allele – see methods. *n* = 3 replicates of > 100 animals per replicate. Error bars – s.d. *** p <0.001, **** p < 0.0001.

If a mother’s environment regulates offspring’s ability to adapt to osmotic stress via changes in oocyte mitochondrial protein composition, then we hypothesized that some changes in mitochondrial protein composition might still be detectable in offspring. To test this hypothesis we performed global proteomics profiling on ~50,000 embryos (n = 8 replicates each) collected from parents that were exposed to normal laboratory conditions (50 mM NaCl), and parents exposed to mild osmotic stress (300 mM NaCl). We detected peptides matching 7,161 *C. elegans* proteins and found that the abundance of 371 proteins was significantly (*padj* < 0.01) changed in the offspring of parents exposed to 300 mM NaCl (Fig. 1a and Supplementary Table 1). These 371 proteins included glycerol-3-phosphate dehydrogenase GPDH-2^3^, the O-GlcNAc transferase OGT-1^5^, and the late embryo abundant protein LEA-1^6^ (Fig. 1a), all of which participate in the osmotic stress response. In addition to these known osmotic stress proteins, we discovered that the majority of regulated proteins were related to animal metabolism and that mitochondria was the most common cellular localization site for differentially abundant proteins (Fig 1b and Supplementary Table 1). Differentially abundant mitochondrial proteins included two subunits of ETC complex II (SDHA-1 and SDHA-2) and two subunits of complex V (ASG-2 and MAI-2; Fig. 1a). Thus, parental exposure to osmotic stress alters the abundance of metabolic and mitochondria-localized proteins in offspring.

When we compared these proteomics data to our previous embryo RNA-seq data (from parents exposed to either 50 mM or 300 mM NaCl^3^), we found that only 106 of the 371 proteins had a corresponding change in mRNA abundance (Supplementary Fig. 1a). It is possible that the observed discrepancy between proteomic and RNA-seq data is an artifact of rapid mRNA degradation of mRNA contributed from oocytes. Thus, we also manually dissected oocytes from wild-type animals exposed to either 50 mM or 300 mM NaCl and performed oocyte RNA-seq to capture “earlier” mRNA expression patterns (Supplementary Fig. 1b). We identified 169 differentially expressed genes in oocytes from parents exposed to osmotic stress (Supplementary Fig. 1c and Supplementary Table 2). However, only 5 of these 169 differentially expressed genes overlapped with changes in protein abundance in embryos (Supplementary Fig. 1d). Interestingly, *gpdh-2* did not exhibit an increase in mRNA abundance in either oocytes or embryos (Supplementary Figure 1)^3^, even though GPDH-2 protein is more abundant in the offspring of parents exposed to 300 mM NaCl (Fig. 1a) and GPDH-2 is functionally required for animals to intergenerationally adapt to osmotic stress^3^. Proteomics therefore identifies functionally relevant changes in offspring protein abundance that cannot be inferred from RNA-seq data alone.

### Oocyte ETC composition affects offspring response to stress

Our proteomics data suggested that changes in mitochondrial ETC composition or function might be required for animals to intergenerationally adapt to osmotic stress. To test this hypothesis, we took *C. elegans* mutants with known point mutations in ETC complex I (*gas-1*^7^, *nuo-6*^8^, *nduf-7*^9^), complex II (*mev-1*^10^, *sdha-2*), complex III (*isp-1*^11^), and complex V (*asg-2*), and assayed whether they could intergenerationally adapt to osmotic stress. As we found previously^3^, >99% of wild-type embryos collected from parents grown under normal laboratory conditions (50 mM NaCl) enter a reversible and stress-resistant state of near suspended animation in response to 500 mM NaCl after hatching (Fig. 1c). This response helps animals survive otherwise lethal exposure to osmotic stress^3^. By contrast, parental exposure to mild osmotic stress (300 mM NaCl) generates offspring that are resistant to osmotic stress, and bypass this state of near suspended animation^3^ (Fig. 1c). Point mutations in *gas-1(fc21), nuo-6(qm200), nduf-7(et19), mev-1(kn1)*, and *isp-1(qm150)* resulted in animals that were unable to adapt to osmotic stress (Fig. 1c). ASG-2 and SDHA-2 proteins were elevated in the offspring of wild-type parents exposed to 300 mM NaCl (Fig. 1a), and we also found that mutations in *asg-2(tm1472)* and *sdha-2(tm1420)* reduced animals capacity to adapt to osmotic stress relative to wild-type offspring from parents exposed to 300 mM NaCl (Fig. 1c). (Note, in *C. elegans* ASG-2 and SDHA-2 are partially redundant with ASG-1 and SDHA-1 respectively). In contrast to mutations in subunits of complexes I, II, III, and IV, we found that mutations in Coenzyme Q synthesis (*clk-1(qm30)*), mitochondrial fission (*drp-1*), and mitochondrial fusion (*eat-3*) genes did not disrupt animal’s ability to adapt to osmotic stress, while a mutation in the mitochondrial fusion gene *fzo-1* slightly enhanced the adaptive response (Fig. 1c). We conclude that proper function of complexes I, II, III, and V of the mitochondrial oxidative phosphorylation (OxPhos) machinery is required for animals to intergenerationally adapt to osmotic stress.

Previous studies found that animals with ETC mutations (*nuo-6* and *isp-1*) are resistant to and survive exposure to 500 mM NaCl better than wild-type animals^12,13^. However, our findings indicate that the same mutant animals cannot bypass entry into a state of near suspended animation in response to 500 mM NaCl even when their parents were exposed to 300 mM NaCl (Fig. 1c). Since we previously found that stress-induced near suspended animation promotes animal survival at 500 mM NaCl^3^, we hypothesized that these two seemingly contradictory results might be because OxPhos dysfunction favors animal entry into a protective suspended animation in response to osmotic stress. If true, then OxPhos mutants should enter suspended animation at NaCl concentrations that are normally tolerated by wild-type animals, in the same way that 100% of insulin receptor (*daf-2*) mutant animals enter suspended animation in response to 300 mM NaCl even though 0% of wild-type animals enter this state under the same conditions^3^. To test this, we exposed wild-type, *nuo-6, nduf-7, gas-1, mev-1*, and *isp-1* mutant animals to 300 mM, 350 mM, and 400 mM NaCl, and quantified the number of mobile and developing animals after 48 hours of exposure to osmotic stress. Consistent with our hypothesis, we found that >50% of *isp-1(qm150)* mutants entered the near suspended animation state at 300 mM NaCl, a condition where 100% of wild-type animals develop normally (Fig 1d). *gas-1(fc21), nduf-7(et19), nuo-6(qm200),* and *mev-1(kn1)* mutants were more prone than wild-type animals to enter developmental arrest in response to 350 mM and 400 mM NaCl, but these effects were relatively subtle compared to *isp-1(qm150)* mutants (Fig. 1e). To rule out that the observed phenotype was due to background mutations in the *isp-1(qm150)* strain, we used CRISPR/Cas9 to recreate the P225S mutation in *isp-1* in wild-type animals, and confirmed that this newly and independently generated mutant behaves the same as *isp-1(qm150)* (Fig. 1f). To test if ETC mutants cause defects in animals’ response to osmotic stress due to a global loss of ATP abundance or due to defects in glycerol metabolism we profiled the abundance of these metabolites in wild-type and *isp-1* mutant embryos from parents exposed to 50 mM and 300 mM NaCl (*isp-1* mutants were the most sensitive to osmotic stress (Fig. 1d-e)). However, we found that the global ATP/ADP ratio was slightly increased in *isp-1* mutant animals and that *isp-1* mutants had no changes in either baseline glycerol abundance or the ability to increase glycerol abundance in response to osmotic stress (Supplementary Figure 2). We conclude that ETC function regulates *C. elegans* decision to enter a protective near suspended animation like state in response to osmotic stress and the effects of ETC dysfunction on animals’ response to osmotic stress are not due to a global loss of ATP abundance or due to a defect in glycerol metabolism.

We previously found that insulin signaling to oocytes regulated whether or not *C. elegans* enter a suspended animation like state in response to osmotic stress^3^. To test if ETC subunits might also function in oocytes to regulate offspring response to osmotic stress we expressed a wild-type copy of *isp-1* specifically in germ cells using the *pie-1* promoter in *isp-1(qm150)* mutant animals. We found that this germ cell specific expression was sufficient to rescue the defects caused by the *isp-1(qm150)* mutation (Fig. 1f). We conclude that altering germline ETC subunit composition is sufficient to alter offspring response to osmotic stress at hatching.

**Figure 2.**
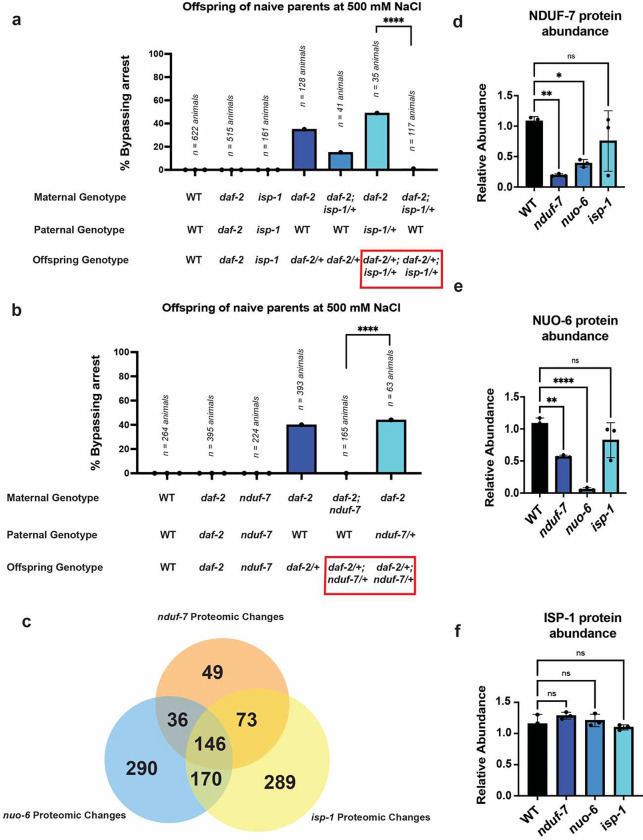
Proper maternal OxPhos composition is required to link maternal insulin signaling to oocytes to offspring response to osmotic stress at hatching. (a) Percent of wild-type, *daf-2(e1370),* and *isp-1(qm150)* mutant animals developing and mobile after 48 hours of exposure to 500 mM NaCl. *daf-2(e1370); isp-1(qm150)/+* animals harbor the *nT1[qIs51]* balancer to maintain heterozygous animals. Cross progeny from heterozygous parents were individually genotyped for each animal, and total animals for each cross are listed. Paternal genotype also contained *him-8(e1489); otIs181* to generate male animals, and confirm cross progeny as previously described^3^. The red box highlights genetically identical offspring that have different responses to osmotic stress. (b) Percent of wild-type, *daf-2(e1370),* and *nduf-7(et19)* mutant animals developing and mobile after 48 hours of exposure to 500 mM NaCl. Cross progeny from heterozygous parents were individually genotyped for each animal, and total animals for each cross are listed. Paternal genotype also contained *him-8(e1489); otIs181* to generate male animals and confirm cross progeny, as previously described^3^. The red box highlights genetically identical offspring that have different responses to osmotic stress. (c) Venn Diagram of proteins that change in abundance by proteomics profiling in *nuo-6(qm200), nduf-7(et19),* and *isp-1(qm150)* mutant embryos. See Supplementary Table 2 for complete list of individual proteins. (d) Relative abundance of NDUF-7 protein in *nuo-6(qm200), nduf-7(et19),* and *isp-1(qm150)* mutant embryos. n = 3 replicates. Error bars – s.d. (e) Relative abundance of NUO-6 protein in *nuo-6(qm200), nduf-7(et19),* and *isp-1(qm150)* mutant embryos. n = 3 replicates. Error bars – s.d. (f) Relative abundance of ISP-1 protein in *nuo-6(qm200), nduf-7(et19),* and *isp-1(qm150)* mutant embryos. n = 3 replicates. Error bars – s.d. * = *p* < 0.05, ** = *p* < 0.01, **** = *p* < 0.0001

If mitochondrial function or composition in oocytes regulates offspring response to osmotic stress after hatching, then disruption of maternal, but not paternal, mitochondrial function should disrupt offspring adaptation to osmotic stress. To test this, we used a series of genetic crosses similar to those we previously used to find that the insulin-like receptor DAF-2 functions in oocytes to regulate *C. elegans* intergenerational adaptation to osmotic stress^3^. Briefly, we previously found that 100% of *daf-2(e1370)* homozygous mutant animals enter a state of near suspended animation in response to even mild osmotic stress (300 mM NaCl – see Fig. 2a and^3^). This sensitivity to developmental arrest is due to a loss of insulin signaling to intestinal cells which drives arrest^3^. However, when *daf-2* mutant mothers are crossed with wild-type males it results in offspring that are resistant to developmental arrest even at 500 mM NaCl (Fig. 2a and^3^). This unusual maternal effect that is distinct from either the maternal or paternal phenotype is because heterozygous *daf-2/+* offspring have functional insulin signaling to intestinal cells which reveals a previously masked maternal effect of reduced insulin signaling to oocytes on offspring response to osmotic stress^3^. In other words, the loss of insulin signaling to oocytes triggers the same intergenerational adaptation to osmotic stress as maternal exposure to 300 mM NaCl even when parents are not exposed to any osmotic stress (See Burton et al., 2017 for details^3^). To test if test if ISP-1 is required maternally to promote offspring adaptation to osmotic stress we first attempted to generate *daf-2(e1370); isp-1(qm150)* homozygous double mutant animals. However, we found that such double mutant animals were sterile. Nonetheless, we were able construct *daf-2(e1370); isp-1(qm150/+)* animals that were heterozygous for the *isp-1(qm150)* mutation. We found that when *daf-2(e1370); isp-1(qm150/+)* mutants were crossed with wild-type males then 99% of offspring that inherited the *isp-1(qm150)* mutation from their mothers arrested their development at 500 mM NaCl (Fig. 2a – column 7). This effect was due to the maternal presence of the *isp-1(qm150)* mutation because genetically identical offspring that inherited the *isp-1(qm150)* mutation from their fathers showed no defect in adapting to 500 mM NaCl (Fig. 2a – column 6). We conclude that the function of maternally expressed *isp-1* regulates offspring response to osmotic stress. Importantly, the offspring enter the state of suspended animation in response to osmotic stress only after hatching. To our knowledge, this is the first example of maternal OxPhos machinery affecting an offspring phenotype that arises *after* completing embryonic development.

To test if our findings for *isp-1(qm150)* were unique to ETC complex III or the *isp-1(qm150)* mutation, we performed analogous crosses and tests with the *nduf-7(et19)* mutants that were most similar to wild-type animals in their naïve response to osmotic stress (Fig. 1f). In this case, *daf-2(e1370); nduf-7(et19)* double mutants were homozygous viable. When these double mutant mothers were crossed with wild-type males, none of the offspring bypassed suspended animation in response to 500 mM NaCl (Fig. 2b, column 5). However, genetically identical offspring that inherited the *nduf-7 (et19)* mutation from the father were able to adapt to 500 mM NaCl (Fig. 2b, column 6). Thus, maternal dysfunction in either ETC complex I (*nduf-7*) or complex III (*isp-1*) blocks the intergenerational adaptation to osmotic stress.

### ETC mutations alter the abundance of diverse mitochondrial proteins

Disruptions in diverse subunits of complexes I, II, III, and V all blocked animals’ ability to intergenerationally adapt to osmotic stress even though these mutations have very different effects on other animal phenotypes like aging. We hypothesized that one possible explanation for this discrepancy is because all of these mutations might themselves alter the abundance of ETC complexes or subunits either directly or indirectly as part of biological mechanisms to compensate for or tolerate mutations in these ETC genes. In this case then any mitochondrial remodeling that occurs to allow toleration of mutations in these diverse ETC subunits might also prevent the normal mitochondrial remodeling in oocytes/embryos that occurs in response to osmotic stress. If true, then we also hypothesized that mutations in these ETC subunits should also result in mitochondrial remodeling whether or not animals are exposed to osmotic stress. To test this hypotheses, we performed unbiased, global proteomics on wild-type, *isp-1(qm150), nuo-6(qm200)*, and *nduf-7(et19)* mutant embryos,. We found that 146 proteins exhibited significant changes in abundance in all three mutant backgrounds relative to wild-type embryos (Fig. 2c). Forty-five of these 146 proteins localize to mitochondria, including multiple subunits of complex I. These findings are consistent with our hypothesis that mutations in diverse ETC subunits all result in substantial changes in the abundance of mitochondrial proteins, either indirectly or as part of a mechanism to compensate for abnormal ETC function. Of the 146 proteins that change in abundance in all three ETC mutants, twenty-four were also among the 371 proteins that exhibited altered abundance in the offspring of parents exposed to 300 mM NaCl (Fig. 1a), suggesting that mutations in ETC subunits also affect the abundance of dozens of proteins that are regulated by osmotic stress (Fig. 1a and Supplementary Table 3). Notably, complex 1 mutations (*nuo-6, nduf-7*) reduced the abundance of most other complex I subunits (including *nuo-6* and *nduf-7,* respectively; Figs. 2d, 2e, and Supplementary Table 3), and 80% of the protein changes in *nduf-7(et19)* mutants were also observed in *nuo-6(qm200)* mutants. By contrast, the *isp-1(qm150)* mutation affected 289 proteins that were not altered in either complex I mutant (*nuo-6, nduf-7*), nor did it alter *isp-1* protein levels (Fig. 2f). We conclude that mutations in diverse ETC subunits change the abundance of many (distinct) mitochondrial proteins, including those that are regulated by maternal exposure to 300 mM NaCl. These findings are consistent with a model in which mutations in diverse ETC subunits all block animals ability to intergenerationally adapt to osmotic stress because they have abnormal compositions of ETC subunits that are part of animals normal adaptation to osmotic stress.

### ETC function modifies animal stress response via AAK-2

**Figure 3.**
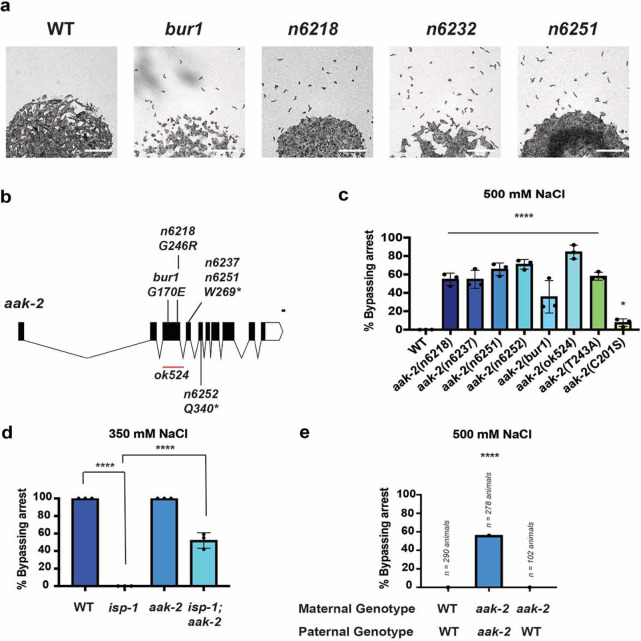
ETC dysfunction regulates *C. elegans* response to osmotic stress via and AMP-kinase/aak-2 dependent mechanism. (a) Representative images of >500 wild-type and *aak-2* mutant *C. elegans* embryos placed on NGM agar plates containing 500 mM NaCl for 48 hours. 100% of wild-type animals enter suspended animation at hatching, while a fraction of *aak-2* mutants bypass suspended animation and continue developing. For imaging purposes, plates contained no food and mobile animals remain in the L1 larval stage. Scale bars 1 mm. (b) Diagram of the *aak-2* mutations recovered from mutagenesis screens. Red bar represents deletion region. (c) Percent of wild-type and *aak-2* mutant animals developing and mobile after 48 hours of exposure to 500 mM NaCl. *n* = 3 replicates of >100 animals per replicate. Error bars – s.d. (d) Percent of wild-type, *aak-2(n6251),* and *isp-1(qm150)* mutants developing and mobile after 24 hours of exposure to 350 mM NaCl. *n* = 3 replicates of >100 animals per replicate. Error bars – s.d. (e) Number of wild-type and *aak-2(n6251)* mutant cross progeny developing and mobile after 48 hours of exposure to 500 mM NaCl. Paternal genotypes also contained *him-8(e1489); otIs181* to generate male animals and confirm cross progeny, as previously described^3^.

To identify additional genes involved in the intergenerational adaptation to osmotic stress, we performed two independent mutagenesis screens. One screen was performed on wild-type animals to identify mutants that could bypass suspended animation when exposed to 500 mM NaCl. The second screen was performed on *isp-1(qm150)* animals to identify suppressor mutants that could bypass suspended animation when exposed to 350 mM NaCl. AMP kinase catalytic subunit *aak-2* alleles were identified from both screens (Fig. 3a-d), and an independent *aak-2(ok529)* deletion allele behaved identically to mutations isolated from our screens (Fig. 3c). AAK-2 is canonically activated *in vivo* by phosphorylating T243^14,15^ (equivalent to mammalian AMPK T172^14–16^) and can be noncanonically activated by oxidation of cysteines by ROS^17,18^. To test if AAK-2 phosphorylation or cysteine oxidation might be required to regulate animal’s response to osmotic stress, we obtained an existing T243A allele of aak-2 generated by CRISPR/Cas9 and, separately, generated a C201S allele of *aak-2* based on the conservation of C201 (in *C. elegans*) across species. We found that T243A mutants behaved like *aak-2* null mutants, while C201S mutants behaved like weak partial loss-of-function mutants (Fig. 3c). We conclude that the canonical phosphorylation site (T243) for activation of AAK-2 is required for AAK-2 to promote entry into a state of near suspended animation in response to osmotic stress. To test if *aak-2* functions maternally or in offspring, we crossed *aak-2(ok529)* mutant mothers with wild-type males, and quantified offspring response to 500 mM NaCl. Offspring from this cross entered suspended animation similar to wild-type animals (Fig. 3e). AMP-kinase activity is well-established to be regulated by changes in ETC activity and these findings support a model in which changes in ETC function regulate *C. elegans* response to osmotic stress by modifying AAK-2 activity in offspring.

### AAK-2 regulates ATP and Glycerol metabolism in response to stress

**Figure 4.**
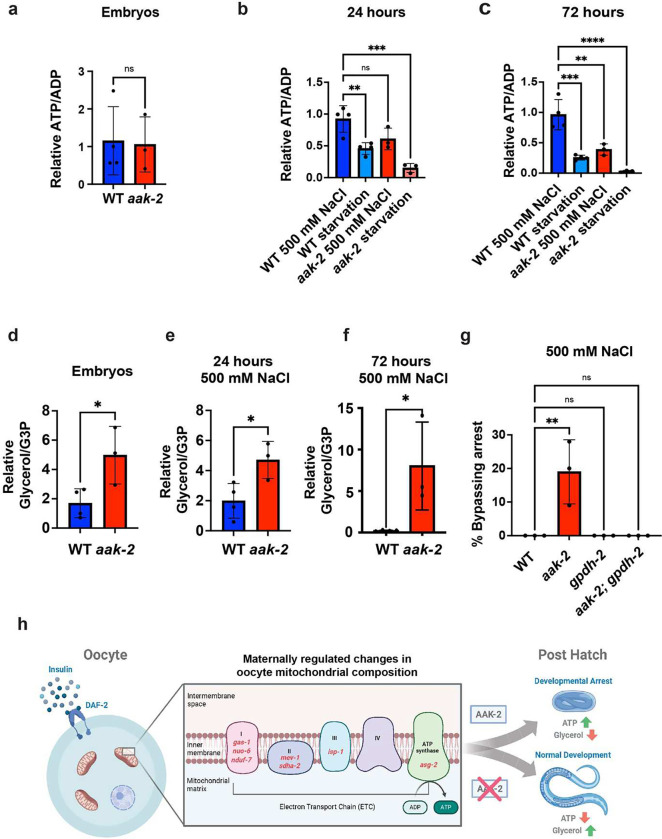
AAK-2 promotes ATP preservation and antagonizes glycerol metabolism to regulate *C. elegans* response to osmotic stress. (a) Relative ATP/ADP ratio (measured by LC/MS) in wild-type and *aak-2(ok524)* mutant embryos. n = 3 replicates. Error bars – s.d. (b) Relative ATP/ADP ratios in wild-type and *aak-2(ok524)* mutant L1 stage animals exposed to 500 mM NaCl (and without food) or starved for 24 hours. n = 3 replicates. Error bars – s.d. (c) Relative ATP/ADP ratios in wild-type and *aak-2(ok524)* mutant L1 stage animals exposed to either 500 mM NaCl (without food) or starved for 72 hours. n = 3 replicates. Error bars – s.d. (d) Relative glycerol/glycerol-3-phosphate (G3P) ratio measured by GC/MS (glycerol) and LC/MS (G3P) in wild-type and *aak-2(ok524)* mutant embryos. n = 3 replicates. Error bars – s.d. (e) Relative glycerol/G3P ratio in wild-type and *aak-2(ok524)* mutant L1 stage animals exposed to either 500 mM NaCl in the absence of food or starvation for 24 hours. n = 3 replicates. Error bars – s.d. (f) Relative glycerol/G3P ratio in wild-type and *aak-2(ok524)* mutant L1 stage animals exposed to either 500 mM NaCl (without food) or starved for 72 hours. n = 3 replicates. Error bars – s.d. (g) Percent of wild-type, *aak-2(n6251),* and *gpdh-2(ok1733)* mutant animals developing and mobile after 24 hours of exposure to 500 mM NaCl. *n* = 3 replicates of >100 animals per replicate. Error bars – s.d. (h) Proposed model for how maternal exposure to osmotic stress influences offspring response to future osmotic stress.

Among its many functions, AMP-kinase activity regulates animal metabolism in response to stress^19^. To better understand how AMP-kinase activity regulates *C. elegans* intergenerational adaptation to osmotic stress, we performed global metabolomics profiling on wild-type and *aak-2(ok529)* mutant animals before exposure to osmotic stress, after 24 hours of exposure to osmotic stress (500 mM NaCl), and after 48 hours of exposure to osmotic stress (500 mM NaCl). Wild-type and *aak-2* mutant starved animals were used as controls to differentiate between metabolic shifts resulting from a general lack of food from those that specifically occur in response to osmotic stress. Two significant findings emerged from these comparisons. First, we found that *aak-2* was required for maintaining ATP levels in response to both osmotic stress and starvation (Fig. 4a-c). This is consistent with the known function of AMP-kinase signaling. Second, glycerol levels were approximately 2 to 8-fold higher in *aak-2* mutants than in wild-type animals (Fig. 4d-f). These data are consistent with our previous results showing that increased glycerol production in offspring promotes resistance to 500 mM NaCl^3^, and indicate that glycerol abundance is regulated by AAK-2 activity. While it is known that glycerol abundance increases in response to osmotic stress^3,20^, we unexpectedly found that animals in suspended animation metabolized most of their glycerol within 72 hours, and did so in an *aak-2* dependent manner (Fig. 4b). These findings suggest that glycerol is more than just an osmolyte that prevents water loss during osmotic stress and is metabolized by animals that have entered a near suspended animation-like state in response to otherwise lethal osmotic conditions.

We previously found that the glycerol-3-phosphate dehydrogenase GPDH-2 is required for animals to adapt to osmotic stress and bypass the near suspended animation like state in response to 500 mM NaCl^3^. To test if *aak-2* mutants also require GPDH-2 to bypass suspended animation in response to 500 mM NaCl, we placed wild-type, *aak-2, gpdh-2*, and *aak-2; gpdh-2* double mutant embryos at 500 mM NaCl and quantified the number of animals that were mobile and developing after 48 hours. Consistent with our previous findings and with *aak-2* mutants having increased glycerol abundance when compared to wild-type animals, we found that *gpdh-2* was required for *aak-2* mutants to develop at 500 mM NaCl (Fig. 4g). We conclude that AAK-2 regulates glycerol abundance and the intergenerational adaptation to osmotic stress via a GPDH-2 dependent mechanism.

In conclusion, our results support a model (Fig. 4h) where maternal exposure to mild osmotic stress causes reduced insulin signaling to oocytes^3^. Reduced insulin signaling to oocytes changes the abundance of mitochondrial and ETC proteins in oocytes (as it does in flies and frogs^1^), which in turn alters offspring AAK-2 activity to promote animals’ metabolic adaptation to osmotic stress. To our knowledge, this is the first demonstration that changes in oocyte mitochondria ETC composition can drive long-lasting changes in offspring phenotype, without any changes in offspring genotype. These findings change our understanding of mitochondrial inheritance; they suggest that oocytes inherit more than just mtDNA from the mother, and that changes in oocyte ETC composition can transmit environmental information to offspring that tailors offspring metabolism to best match the environment experienced by their mothers.

Intergenerational and transgenerational responses to environmental stress have been reported in diverse species^3,21–38^. Mechanistic follow up studies of these findings to date have largely focused on epigenetic or small RNA based mechanisms (reviewed in^39^). Here, we report a largely novel mechanism regulating an intergenerational adaptation to stress, the transmission of altered mitochondria to offspring via oocytes. This mechanism would neatly explain multiple unanswered questions in the field of intergenerational effects including: Why do some of the most robust known models of intergenerational adaptations to stress (>10-fold increases in offspring survival) only transmit maternally but not paternally^3,21,22,24,40^? And how can environmental information be maintained through gametogenesis and early embryogenesis when there is extensive epigenetic remodeling^41^ ?

The complete extent to which insulin signaling to oocytes and mitochondrial proteome remodeling in oocytes regulates offspring metabolism across species, including in humans, remains unknown. However, the fact that oocyte mitochondrial remodeling in oocytes continues to be identified in all taxa investigated to date, including in humans^2^, suggests that is potentially an evolutionarily ancient phenomena that plays a major role in animal fitness and physiology. Interestingly, recent studies of *D. melanogaster* found that reduced insulin signaling to oocytes increases offspring’s resistance to nutrient stress via a mechanism that depends on changes in the NAD^+^/NADH ratio^4^. Coupled with our findings in *C. elegans*, these two studies indicate that insulin signaling to oocytes regulates two distinct intergenerational responses to stress (osmotic, nutrient) across two diverse taxa (*C. elegans* and *D. melanogaster*)^3,4^. While this general mechanism was the same across these two species, we found that the *C. elegans* adaptation to osmotic stress is mediated by an AMP-kinase dependent mechanism which is normally regulated by changes in the ratio of ATP compared to ADP and AMP. By contrast, the *D. melanogaster* response to nutrient stress appears to be regulated by a different metabolic ratio, the NAD^+^/NADH ratio^4^. This difference suggests the exciting possibility that mitochondrial remodeling in oocytes might be a tunable mechanism that responds to many different environmental inputs across species and that it might drive different and tailored metabolic responses in offspring that tune offspring metabolism to maximize fitness in the environment experienced by their mothers – *i.e.* the environment in which they are about to be born into. Future studies of this phenomena will be critical in determining if oocyte mitochondrial remodeling is in fact tunable in response to different stimuli and the extent to which it impacts offspring metabolism across species. Nonetheless, because a similar mitochondrial remodeling event occurs in human oocytes^2^, these results might help explain why and individual’s risk for metabolic disease (e.g., Type 2 diabetes) has already been epidemiologically linked to a mother’s environment^42,43^.

## Methods

### Strains.

*C. elegans* strains were cultured and maintained at 20 °C unless noted otherwise. The Bristol strain N2 was the wild-type strain. Mutations used in this study: *nuo-6(qm200), nudf-7(et19), gas-1(fc21), mev-1(kn1), sdha-2(tm1420), isp-1(qm150), asg-2(tm1472), clk-1(qm30), fzo-1(tm1133), eat-3(ad426), drp-1(tm1108), daf-2(e1370), aak-2(n6218, n6237, n6251, n6252, bur1, ok524, jbm54[C201S], and stj17[T243A]), him-8(e1489).* The *nT1[qIs51]* balancer was used to maintain *isp-1(qm150)* as a heterozygote in *daf-2(e1370); isp-1(qm150/+). otIs181* was used to confirm cross progeny in experimental crosses as reported previously (reference #3). *Isp-1(syb7165)* is a CRISPR generated recreation of the P225S mutation found in *isp-1(qm150).* sybIs7373 encodes *[Ppie-1-nuo-6 gDNA-tbb-2 3’UTR]* to rescue ISP-1 function specifically in germ cells.

### Offspring embryo isolation.

Animals grown in normal NGM conditions were collected in M9 and then washed 3x in diH_2_0. Washed animals were then treated with a solution of of 5% sodium hypochlorite and 5 M NaOH,for 5 minutes under agitation. The embryos were then collected via centrifugation and treated with a secondary solution of 10% sodium hypochlorite for less than 1 minute and observed at 4x magnification until remaining detritus was degraded. Eggs were then collected and washed 3x in M9 if the eggs were to be utilized is osmotic stress assays or washed 3x in diH_2_O if eggs were to be utilized in proteomic or metabolomics analysis.

### Osmotic stress assay.

Adult animals raised under normal NGM conditions were collected in M9 buffer solution and embryos were isolated utilizing our embryo isolation protocol (see above). Eggs were then transferred to osmotically stressful culture plates at various concentrations (300, 350, and 400mM NaCl) and incubated at room temperature for 48 hours. Hatched were then quantified and removed utilizing vacuum suction. Arrested animals were then collected using M9 buffer and allowed to recover in normal NGM culture conditions for 24 hours and then quantified. Osmotic stress assays using 500mM NaCl culture conditions were incubated for only 24 hours before quantification and recovery.

### Adaptation assay.

Adolescent mothers (at the L4 larval stage) were transferred in M9 buffer solution from normal NGM cultures to osmotically stressful 300mM NaCl cultures until animals were gravid (approximately 24 hours). After which time the cultures were collected and eggs were isolated using our egg isolation protocol. Harvested eggs were then transferred to 500mM NaCl cultures. After 48 hours hatched animals were quantified and removed utilizing vacuum suction and arrested embryos were collected in M9 buffer solution and allowed to recover in normal NGM culture conditions for 24 hours after which time recovered animals which regain normal activity were quantified.

### Experimental crosses.

To generate heterozygous mutant strains genetic crosses were performed. Adolescent mothers (at the L4 larval stage) from the parental strains were mated with males from the desired strains on NGM culture agar plates for 24–48 hours. The parent carrying the mutations were changed from maternal to paternal depending on the experimental design. Note that mitochondrial mutant males do not mate efficiently, and mutant mothers do not generate large brood sizes. To ensure successful mating males carrying fluorescent tags were ensure that progeny was actually produced from a mating pair. Laid eggs were then individually selected and transferred to 500mM NaCl cultures and allowed to develop at room temperature for 48 hours and the percentage of hatched animals was quantified.

### Proteomics.

*C.elegans* samples were homogenized on the Bead Ruptor Elite (Cat# 19–042E, Omni International) for 30 s in 4% SDS solution containing 1x HALT Protease (Cat# 78442, Thermo Fisher Scientific). Samples were sonicated and clarified via centrifugation and transferred to a Protein LoBind Eppendorf tube. Proteins were quantified using the Pierce BCA Protein Assay Kit (Cat# 23227, Thermo Fisher Scientific) and 100 μg of protein was aliquoted for digestion. Protein digestion utilized the S-Trap (Cat# CO2-Mini, Protifi) platform to remove any SDS prior to LCMS/MS analysis. Briefly proteins were reduced with Dithiothreitol (DTT) for 20 minutes, alkylated with Iodoacetamide (IAA) for 20 minutes and digested overnight with Trypsin/Lys-C (Cat# V5072, Promega) at a ratio of 50:1 (protein:enzyme (w/w)). Peptides were eluted off the S-Trap Column and dried down in a Genevac SpeedVac. Dried samples were then cleaned up with C-18 reverse phase columns from Harvard Apparatus(Cat# 74–4601) and dried down before resuspension for LC-MS/MS analysis. Dried samples were resuspended in 50 μL 0.1% FA (LS118–1, Fisher Scientific) and diluted with 50 μL of 0.1% TFA (LS119–500, Fisher Scientific).

Data Independent acquisition analyses were performed on Orbitrap Eclipse coupled to Vanquish Neo system (Thermo Fisher Scientific). The FAIMS Pro source (Thermo Fisher Scientific) was located between the nanoESI source and the mass spectrometer. 2 μg of digested peptides were separated on a nano capillary column (20 cm × 75 μm I.D., 365 μm O.D., 1.7 μm C18, CoAnn Technologies, Washington, # HEB07502001718IWF) at 300 nL/min. Mobile phase A consisted of LC/MS grade H2O (LS118– 500, Fisher Scientific), mobile phase B consisted of 20% LC/MS grade and H2O and 80% LC/MS grade acetonitrile (LS122500, Fisher Scientific), and both mobile phases contained 0.1% formic acid. The LC gradient was: 1% B to 24% B in 110 min, 85% B in 5 min, and 98% B for 5 min, with a total gradient length of 120 min. For FAIMS, the selected compensation voltage (CV) was applied (−40V, −55V, −70V) throughout the LC-MS/MS runs. Full MS spectra were collected at 120,000 resolution (full width half-maximum; FWHM), and MS2 spectra at 30,000 resolutions (FMWH). Both the standard automatic gain control (AGC) target and the automatic maximum injection time were selected. A precursor range of 380–980 m/z was set for MS2 scans, and an isolation window of 50 m/z was chosen with a 1 m/z overlap for each scan cycle. 32% HCD collision energy was used for MS2 fragmentation. To generate a hybrid library for directDIA^™^ analysis in Spectronaut, pooled samples underwent data-dependent acquisition employing 11 distinct FAIMS CV settings ranging from −30 to 80 CV. Full MS spectra were collected at 120,000 resolution (full width half-maximum; FWHM), and MS2 spectra at 30,000 resolutions (FMWH). Both the standard automatic gain control (AGC) target and the automatic maximum injection time were selected. Ions were filtered with charge 2–5. An isolation window of 1.6m/z was used with quadruple isolation mode. Ions were fragmented using higher-energy collisional dissociation (HCD) with a collision energy of 30%.

DIA data was processed in Spectronaut (version 18, Biognosys, Switzerland) using direct DIA. Data was searched against of Caenorhabditis elegans reference proteome including both UniProt and TrEMBL databases. The manufacturer’s default parameters were used. Briefly, trypsin/P was set as digestion enzyme and two missed cleavages were allowed. Cysteine carbamidomethylation was set as fixed modification, and methionine oxidation and protein N-terminus acetylation as variable modifications. Identification was performed using a 1% q-value cutoff on precursor and protein levels. Both peptide precursors and protein false discovery rate (FDR) were controlled at 1%. Ion chromatograms of fragment ions were used for quantification. For each targeted ion, the area under the curve between the XIC peak boundaries was calculated. To enhance proteome coverage, the DDA raw files were utilized in Library Extension Runs to generate a hybrid library.

### LIMMA Proteomics analysis.

Differential abundance of proteins was performed using LIMMA^44^ and eBayes tools. For differential abundance of proteomics based on 8 replicates of samples (Fig. 1a), proteins with 0 variance and proteins with >30% missingness in any experimental group were removed from analysis. We examined the remaining missingness in the dataset and found that proteins that had missing values in any samples had lower abundance values than proteins without any missingness. Since the missing values were not missing at random, we used a left-censored imputation method (impute.QRILC). Data were then log2 transformed to prepare for LIMMA analysis. We next used LIMMA eBayes to analyze the log2 transformed data with custom contrasts. Both LIMMA and empirical Bayes were performed using robust methods to avoid one protein or sample having too much influence over the results. The results were multiple testing corrected using the Benjamini-Hochberg method to maintain a 5% false discovery rate.

For proteomics comparisons based on 3 biological replicates (Fig. 3), only proteins that exhibited zero variance across all samples were also removed from the analysis. The remaining data were then transformed via variance normalization stabilization to prepare for the LIMMA eBayes analysis. We next used LIMMA eBayes to analyze the normalized data with custom contrasts. Both LIMMA and empirical Bayes were performed using robust methods to avoid one protein or sample having too much influence over the results. The results were multiple testing corrected using the Benjamini-Hochberg method to maintain a 5% false discovery rate.

### Metabolomics.

Metabolites were extracted from embryos using a modified Bligh-Dyer. Briefly, embryos were diluted to 310μL in ice-cold water, to which 690μL of ice-cold chloroform:methanol (1:1, v/v) was added. The sample was vortexed, sonicated for 5 minutes in a water-bath sonicator, and incubated on wet ice for 40 minutes. Samples were then centrifuged at 14,000×g for 10 min at 4C. 450μL of the upper, aqueous layer was collected and dried in a vacuum evaporator. Of the remaining aqueous layer, 25–50 μL of each sample was collected and pooled to serve as a pooled quality control sample in LCMS and GCMS analysis. Dried extracts were resuspended in 45μL of LCMS grade water containing 0.5ug/mL D5 glutamate (DLM-556, Cambridge Isotopes) as an internal standard.

Metabolomics data were collected on an Orbitrap Exploris 240 (Thermo) using a tributylamine ion-paired reversed phase chromatography (PMID: 37095747). 2μL of resuspended extracted were injected on the LC column (ZORBAX Rapid Resolution HD; 2.1 × 150 mm, 1.8 μm; 759700–902, Agilent). Mobile phase A was 3% methanol, and mobile phase B was 100% methanol, each containing mM tributylamine (90780, SigmaAldrich, St Louis, MO, USA), 15 mM acetic acid and 2.5 μM medronic acid (5191–4506, Agilent Technologies, Santa Clara, CA, USA). The LC gradient was: 0–2.5 min 0% B, 2.5–7.5 min ramp to 20% B, 7.5–13 min ramp to 45% B, 13–20 min ramp to 99% B, 20–24 min hold at 99% B. Flow rate was 0.25 mL/min, and the column compartment was heated to 35°C. The column was then backflushed with 100% acetonitrile for 4 min (ramp from 0.25 to 0.8 mL/min in 1.5 min) and re-equilibrated with mobile phase A for 5 min at 0.4 mL/min. The H-ESI source was operated at spray voltage of −2500 V, sheath gas: 60 a.u., aux gas: 19 a.u., sweep gas: 1 a.u., ion transfer tube: 300°C, vaporizer: 250°C. Full scan MS1 data was collected from 70 to 800 m/z at mass resolution of 240,000 FWHM with RF lens at 35%, and standard automatic gain control (AGC). ddMS2 data were collected on pooled samples with MS1 resolution at 60K, intensity threshold at 2.0 × 104, dynamic precursor exclusion for 10s, and MS2 resolution at 30K with stepped collision energies at 20, 35, and 50%. Data were analyzed in Skyline (v23.1) using a compound list curated from standard verified retention time, accurate mass MS1 (+/− 5ppm), and MS2 fragmentation spectra.

For measurement of glycerol, samples were dried post LCMS analysis and subjected to derivatization with 30 μL of methoxyamine (11.4 mg/mL) in pyridine and 70 μL of MTBSFA+1%TMCS TBDMS for GCMS analysis on an Agilent 7890GC/5977bMSD as described previously (PMID: 35981545, PMID: 31747582). The oven program was: initial 95°C ramp to 118°C at 40°C/min then hold for 2 min, then ramp to 300°C at 12°C/min, and hold at 300°C for 5 min. A neat standard of glycerol was derivatized and used to determine retention time (10.77min) and fragment ions (170.8, 188.7, 289, and 377). 170.8m/z was used as the quantifier ion, and others used to qualify the peak. Data were analyzed in Skyline.

### Oocyte dissections

Animals were raised to adolescence (L4 stage) under normal culture conditions on NGM and then split into separate cultures, control or 300mM NaCl media, until day 1 of adulthood. Animals were then collected in M9 buffer and washed 3x. Single animals were then transferred to a microscope slide and dissected in a 10uL drop of paralytic 0.5 mg/mL tetramisole in M9. The procedure was performed using a dissection microscope at 4x magnification. The first incision was performed using a number 11 surgical scalpel on the paralyzed animal just posterior to the pharynx, exposing the intestine and the anterior gonad arm. The exposed gonad arm was then removed utilizing a 25 G × 5/8 needle and transferred to a 10uL solution of pure M9. The −1, −2, and −3 oocytes were then separated from the gonad arm using a 34 gauge RN needle, resulting the separation of the oocytes connected together with a small amount of gonadal sheath. These cells were then transferred via eyelash pick into 8uL of SMART seq solution and frozen at −80 C.

### Oocyte RNA-seq and analysis.

RNA was prepped from oocytes using the Direct-zol RNA microprep kit (ZYMO Research) and was sequenced using an Illumina NovaSeq 6000. Reads were aligned to the WBcel235 reference genome and quantified with STARsolo. A gene matrix composed of a row for each gene and column for each sample was built and brought into R using SummarizedExperiment. Genes with fewer than ten reads across all samples were removed. Differential analysis of the quantified genes was performed with DESeq2^45^.

### Statistical analysis.

ANOVA analysis with post hoc p-value calculations was used for Figs. 1c, 1d, 1e, 1f, 2d, 2e, 2f, 3c, 3d, 4b, 4c, 4g, and Supplementary Fig. 2b. Fisher’s exact test was used for Figs. 2a, 2b, and 3e. Unpaired two-tailed Student’s t-test was used for Figs. 4a, 4d, 4e, 4f, and Supplementary Fig. 2a. LIMMA analysis for global proteomics (Fig. 1a) and DESeq2 analysis for RNA-seq (Supplementary Fig. 1) are described above in separate sections. No statistical method was used to predetermine sample size. The experiments were not randomized. The investigators were not blinded to allocation during experiments and outcome assessment.

## Supplementary Material

Supplement 1

## Data Availability

RNA-seq data that support the findings of this study have been deposited at NCBI GEO and are available under the accession code GSE261225. Proteomics data that support the findings of this study have been deposited in the PRIDE repository and are available under the accession code PXD050192.
