# Supplementary material for: Oocyte mitochondria link maternal environment to offspring phenotype
Source: Res Sq. 2024 Mar 29:rs.3.rs-4087193. Preprint. [Version 1] doi: 10.21203/rs.3.rs-4087193/v1 (PMC10996803; doi:10.21203/rs.3.rs-4087193/v1)
Supplement: Supplement 1 [file NIHPPrs4087193v1-supplement-1.pdf]

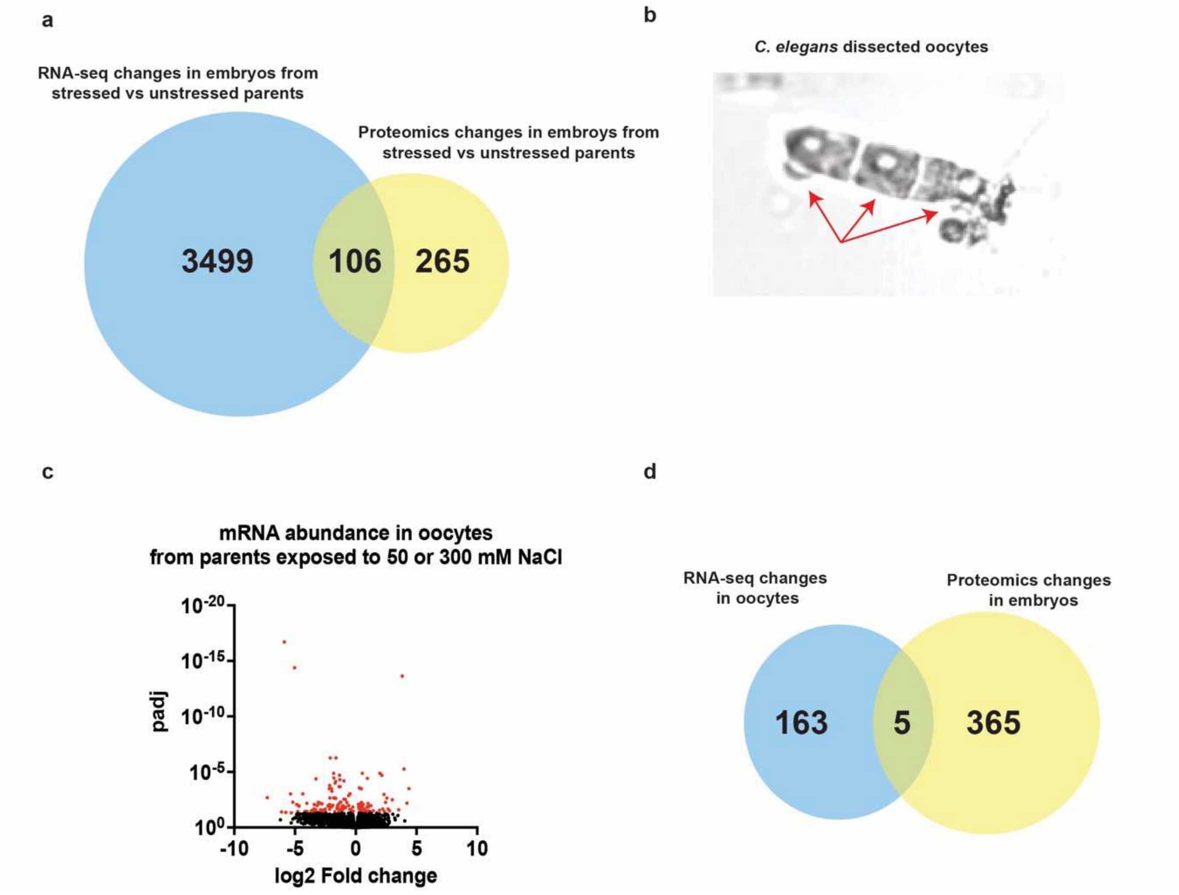

Supplementary Figure 1. Comparison of proteomics and RNA-seq in offspring from parents exposed to osmotic stress. (a) Overlap between detected changes in protein abundance (this study) and differentially expressed genes from Burton et al. 2017. (b) Representative image of three dissected oocytes from *C. elegans* (red arrows). A small amount of sheath remains attached to the oocytes. (c) Volcano plot of differentially expressed genes in dissected oocytes from animals exposed to either 50 mM or 300 mM NaCl for 24 hours. Red dots represent statistically significant changes in abundance ($padj$ < 0.01). (d) Overlap between detected changes in protein abundance and differentially expressed genes in dissected oocytes (from panel (b)) from parents exposed to either 50 mM or 300 mM NaCl for 24 hours.

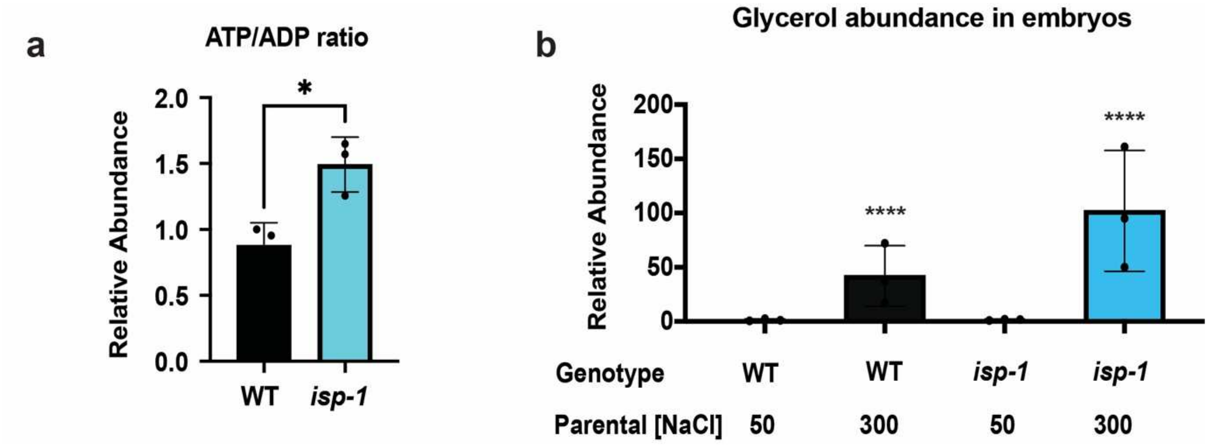

Supplementary Figure 2. The *isp-1(qm150)* mutation did not decrease global ATP abundance or cause defects in glycerol metabolism in response to osmotic stress. (a) Relative ATP/ADP ratio in wild-type and *isp-1(qm150)* mutant embryos as measured by LC/MS. *n* = 3 replicates. Error bars – s.d. (b) Relative glycerol abundance in wild-type and *isp-1(qm150)* mutant embryos as measured by GC/MS. *n* = 3 replicates. Error bars – s.d.

Supplementary Table 1. Global protein abundance in embryos from parents exposed to 50 mM or 300 mM NaCl. Average fold change and statistical significance for 7,161 proteins detected by global proteomics in embryos from parents exposed to 50 mM or 300 mM NaCl. 8 replicates per condition.

Supplementary Table 2. Global mRNA abundance in oocytes from adults exposed to 50 mM or 300 mM NaCl. Average fold change in mRNA abundance for all genes detected in oocytes dissected from adults exposed to either 50 mM or 300 mM NaCl for 24 hours. Three oocytes were dissected from one animal per replicate. Three replicates per condition.

Supplementary Table 3. Global protein abundance in wild-type and ETC mutant animals. Average fold change and statistical significance of all proteins detected in wild-type, *nuo-6(qm200), nduf-7(et19),* and *isp-1(qm150)* mutant embryos. Three replicates per condition.

Statistics Source Data. Source data for all figures.
